# Patagonin-CRISP: Antimicrobial Activity and Source of Antimicrobial Molecules in Duvernoy’s Gland Secretion (*Philodryas patagoniensis* Snake)

**DOI:** 10.3389/fphar.2020.586705

**Published:** 2021-02-02

**Authors:** Juliana Cuoco Badari, Andrea Díaz-Roa, Marisa Maria Teixeira Rocha, Ronaldo Zucatelli Mendonça, Pedro Ismael da Silva Junior

**Affiliations:** ^1^ Laboratory of Parasitology, Butantan Institute, São Paulo, Brazil; ^2^ Laboratory for Applied Toxinology (LETA) ‐ Center of Toxins, Immune-Response and Cell Signaling – CeTICS/CEPID Butantan Institute, São Paulo, Brazil; ^3^ Escuela de Ciencias Agrícolas, Pecuarias y del Medio Ambiente (ECAPMA), Universidad Nacional Abierta y a Distancia (UNAD), Bogotá, Colombia; ^4^ Laboratory of Herpetology, Butantan Institute, São Paulo, Brazil

**Keywords:** CRISP, antimicrobial, bioprospecting, dipsadidae, snakes, toxin and patagonin-CRISP

## Abstract

Snake venom contains a variety of toxins with a range of biological activity, among these toxins cysteine-rich secreted proteins (CRISPs) can be found. The proteins of this family have masses of 20–30 kDa and display homologous amino acid sequences containing 16 cysteine residues, forming eight disulfide bonds. Some of these proteins have been explored, characterized, and described in terms of their activity; however, little is known about their range of activities. A search for new antimicrobial molecules is ongoing, as the number of microbial strains resistant to available antibiotics is increasing. We identified antimicrobial activity in the secretion of Duvernoy's gland of the rear-fanged *Philodryas patagoniensis*. Fractions of this venom were subjected to reverse-phase high performance liquid chromatography and analyzed to determine their antimicrobial activity with a liquid broth inhibition assay. One of the fractions presented activity against a Gram-negative bacterium and a filamentous fungus. This fraction was analyzed with LC-MS/MS, and a protein of 24,848.8 Da was identified. Database searches allowed us to identify it as a CRISP due to the presence of some unique fragments in the molecule. We called it patagonin-CRISP, as the same protein in the venom of *P*. *patagoniensis* had previously been characterized as having a different biological activity. Patagonin-CRISP presented activity at very low concentrations and showed no cytotoxic activity. This is the first time that antimicrobial activity has been identified for *P*. *patagoniensis* venom or for a CRISP family protein.

## Introduction

Microbial resistance is a major public health concern. Pathogenic microorganisms are responsible for countless deaths and generate huge financial losses worldwide. The increased resistance of microorganisms to antimicrobial agents is a serious public health problem that has aroused the attention of the pharmaceutical industry and led it to search for new types of antibiotics. The failure of available antibiotics to contain certain pathogens indicates the need to develop more efficient and less toxic molecules from natural sources (Potron et al., 2015; Vassilara et al., 2017).

Brazil features a wealth of biomes that include a large part of the world's biodiversity; it is considered to have the highest number of endemic species (O'Driscoll et al., 2013), and one cannot deny its proliferation of natural products. These products have been extensively studied and much work have been done on different compounds of biological origin that may be usable in the development of new drugs and to improve existing diagnostic methodologies (Peichoto et al., 2007; Doley and Kini, 2009; Figueroa-Espinoza et al., 2015).

Studies have found antimicrobial activity in snake venom. This activity is linked to the need for self-protective mechanisms against pathogens present in their prey that are triggered by the innate immune system (Thomas and Pough, 1979; Osipov et al., 2005; Perumal Samy et al., 2017).

Venom from rear-fanged snakes, given their complexity and great pharmacological potential, is an interesting category to explore (Fry, 2005; Peichoto et al., 2011).

*Philodryas patagoniensis* is an opisthoglyphous snake, a member of the family Dipsadidae (Utkin, 2015). These snakes are distributed around all South America, inhabiting different environments, and they show high plasticity when adapting to places altered by man in addition to having an excellent ability to camouflage themselves due to their coloration (de Medeiros et al., 2010; Gouveia et al., 2017).

These snakes have a well-developed Duvernoy's gland, connected to their grooved teeth, which, although it is homologous to the venom glands of Solenoglyphous and Proteroglyphous snakes, are functionally and anatomically different (Kardong, 2002; Jackson and Vidal, 2019). Due to its musculature and lack of storage space, this gland produces a low amount of venom, which makes it challenging to study these materials, as it is difficult to obtain sufficient material to characterize it (Modahl and Mackessy, 2019).

Duvernoy's gland has an immediate function in feeding due to the presence of its serous cells, which, with the other components present in the toxic secretion, helps immobilize its prey, lubricates the food, and plays roles in digestive functions, antiputrefaction of the food, microbial neutralization, and oral hygiene (Taub, 1966; Serapicos and Merusse, 2006; Shivik and Carrion, 2009; Oliveira et al., 2016; Weinstein, 2016). The envenomation frame caused by *P*. *patagoniensis* includes local pain, swelling, erythema, ecchymosis, and regional lymphadenopathy with normal coagulation (Mosmann, 1983; Eberspaecher et al., 1995). These symptoms are often confused and treated as bothropic accidents (Silveira and Nishioka, 1992; Ribeiro et al., 1999).

The composition and biological activities of *P. patagoniensis* venom have previously been studied, and some of its components have been isolated and characterized (Hill and Mackessy, 2000; Peichoto et al., 2009; Peichoto et al., 2011; Peichoto et al., 2012).

One of these molecules is a cysteine-rich secretory protein (CRISP) called patagonin (Peichoto et al., 2012). CRISPs are a large family of single-chain bioactive polypeptides with a molecular mass between 20 and 30 kDa. The name of the group comes from the set of cysteine residues settled in its C-terminal region, establishing a compact domain (Eberspaecher et al., 1995). Cysteine-rich venom proteins (CRVPs) are widely distributed among lizard and snake venom (Adade et al., 2014).

The main characteristic here is the homology of the amino acid sequences, which contains 16 highly conserved cysteine residues, which form eight disulfide bonds; 10 of these Cys residues are in the C-terminal portion. In addition to the similarity of their molecular mass and the presence of Cys residues as distinctive structural features, they also have two distinct domains (PR-1 and CRD) (Tadokoro et al., 2020).

The C-terminal region includes the cysteine-rich domain (CRD), which is responsible for the interaction of CRISPs with the ionic channels. The sequence that makes up the N-terminal region is the most conserved among these proteins and belongs to the PR-1 domain. This domain promotes the interaction of these proteins with the target cell (Lima et al., 2006; Gibbs et al., 2008; Estrella et al., 2011; Tadokoro et al., 2020).

The biological activity of most snake venom CRISPs has not yet been described, and their role in envenomation is still uncertain; some studies suggest that they act as ion channel blockers (Silveira and Nishioka, 1992; Osipov et al., 2005; Utkin, 2015; Bernardes et al., 2019). Patagonin has already been characterized, and its biological activity has been described; it expresses necrotic activity in the murine gastrocnemius muscle when injected intramuscularly and has no effect on human platelet aggregation or proteolytic activity (Peichoto et al., 2009).

The CRISP Natrin, isolated from the venom of the snake *Naja atra*, promotes the regulation of the expression of adhesion molecules in endothelial cells that are involved in inflammation (Wang et al., 2010; Samy et al., 2014). Nk-CRISP is a CRVP isolated from the venom of another species of Naja that up regulates inflammatory gene expression in human macrophages (Deka et al., 2020).

Crovirin, isolated from *Crotalus viridis* venom, has been identified to have antiparasitic activity against different strains of Leishmania and Trypanosomes (Adade et al., 2014). The first CRISP from *Bothrops jararaca* has been isolated and denominated Bj-CRP, and it demonstrates possible action on the induction of inflammatory response (Levinskaite, 2012). A CRVP from the *Bothrops alternatus* venom was characterized as having effects on potassium channels and in inflammatory processes. This molecule was named BaltCRP (Bernardes et al., 2019).

The main objective of this study was to isolate, identify, and characterize at a primary structural level the antimicrobial molecules present in Duvernoy's gland secretion of *Philodryas patagoniensis*.

## Materials and Methods

### Venom Samples

The venom was obtained from an adult female belonging to the Zoological Park Foundation of São Paulo. The extraction was performed using a pair of 100 µL microcapillary tubes placed in contact with the rear-fangs according to the procedure previously described (Ferlan et al., 1983). During the extractions, the snake was not anesthetized, and no parasympathomimetic stimulants were used. In all, 16 extractions were performed, and each extraction had a variable yield between 30 and 60 µL.

### Determination of Protein Concentration

The quantification of the samples was performed using Bradford's (1976) method, following the procedure described in the BIO-RAD protocol, with known concentrations of bovine albumin (Sigma) used as the standard.

### Fractionation

Freeze-dried crude *P. patagoniensis* venom (∼2 mg/ml) was homogenized in a 0.05% aqueous solution of TFA-trifluoroacetic acid and subjected to the reverse-phase high performance liquid chromatography (RP-HPLC) Shimadzu LC-10 system. In the first step, the sample was used for semipreparative Jupiter® RP-C18 LC-Column (10 μm, 300 Å, 10 mm × 129 250 mm, Phenomenex™) that had previously been balanced with A solvent (0.05% TFA). Elution was performed in a linear acetonitrile (ACN) gradient equilibrated in 0.05% TFA of 0–80% for 80 min at a flow rate of 2 ml/min. The absorbance was monitored at 225 and 280 nm, and the fractions were manually collected. Then, lyophilized fractions were suspended in 500 µL ultrapure water and an aseptically filtered in Millipore® 0.22 μm filters for antimicrobial assay.

The molecule here described (fraction 33) was submitted to a second step of fractionation in a linear gradient of ACN (20–55%) with 0.05% TFA for 80 min, at a flow rate of 1.0 ml/min and an analytic column Jupiter® C18 LC-Column (10 μm, 300 Å, 4.60 × 250 mm, Phenomenex™), with ACN equilibrated in 0.05% TFA. The hand-collected fractions were dried (Savant Instruments Inc.®) and suspended in 500 µL ultrapure water for MIC and MBC.

### SDS-PAGE

Samples were submitted to 20% SDS-PAGE gel (sodium dodecyl sulfate-polyacrylamide gel electrophoresis). Then, 20 µg protein, solubilized in 20 µL ultrapure water and a sample buffer, was submitted to electrophoresis under reducing conditions. SeeBlue Prestained (Life Technologies R, 198–3 kDa) was used as a molecular mass marker. The proteins were stained with Coomassie Brilliant Blue R-250 (Laemmli, 1970) and Silver (Morrissey, 1981), and the gel images were captured with a photo documentation apparatus (UVITEC, Cambridge, United Kingdom). The protein band of interest was excised from the gel and eluted for antimicrobial activity and mass spectrometry analyses. The same band was digested with trypsin (Hanna et al., 2000) and analyzed by mass spectrometry.

#### Enzymatic In-Gel Digestion

The patagonin-CRISP bands were cut and stored separately in 1.5 ml microtubes. The band was destained in 0.5 ml 50% methanol/5% acetic acid overnight at room temperature before dehydration in 200 μL ACN, followed by complete drying in a vacuum centrifuge. The protein was reduced by adding 50 µL Dithiothreitol-DTT (10 mM) and alkylated by adding 50 µL iodoacetamide-IAA (100 mM) for 30 min at room temperature.

The bands underwent dehydration and rehydration using 200 µL ACN and 200 µL ammonium bicarbonate (100 mM), respectively. Finally, the bands were rehydrated in 50 µL with 20 ng μL^−1^ ice-cold porcine trypsin (Sigma-Aldrich®). Digestion was performed overnight at 37°C. The tryptic peptides produced were collected with successive extractions in 50 µL with 50 mM ammonium bicarbonate) and 50 µL with 50% ACN/5% formic acid (twice). The supernatant was concentrated in a vacuum centrifuge.

### Mass Spectrometry

The samples were suspended in 10 µL 0.1% formic acid solution and analyzed by liquid chromatography coupled to tandem mass spectrometry (LC-MS/MS) using LTQ-Orbitrap Velos equipment (Thermo Fisher Scientific Inc.) coupled to the Easy-nLC II liquid nano-chromatography system (Thermo Fisher Scientific Inc.). A 5 µL sample was automatically injected into a C-18Jupiter C-18 (10 µm × 100 µm × 50 mm) precolumn (Phenomenex), coupled to an ACQUAC-18 analytical reverse phase column (5 µm × 75 µm × 100 mm) (Phenomenex). A linear gradient of 0–60% ACN for 60 min was used, with a flow rate of 200 nL/min. The ionization adopted was used in a positive mode that detects positively charged ions. The mass spectrometry proteomics data have been deposited to the ProteomeXchange Consortium via the PRIDE partner repository with the dataset identifier PXD022842.

### Bioinformatics Analysis

The data were acquired by the Qual Browser program in XCalibur software (version 2.0; Thermo Fisher Scientific), using the default parameters, and the data were exported as text. The intact mass of protein was calculated with the deconvolution of m/z values from the MS spectra text file, performed with the software MagTran 1.02 (Zhang and Marshall,1998;Potron et al.,2015). The MagTran used a mass range of 3,000–30,000 and a charge range of 2–30. The raw data files were processed with a search on PEAKS Studio software (version 8.5, Bioinformatics Solutions Inc.). The analyses had a 10 ppm error tolerance for the precursor ions and a 0.6 Da tolerance for the fragment ions. Oxidation was considered a variable modification, and trypsin was considered to be enzyme-specific. The data were analyzed with cysteine carbamidomethylation as fixed modification and the oxidation of methionine as a variable modification. The MS/MS spectra were searched against the snakes, Dipsadidae, Colubridae, and CRVP databases (downloaded in FASTA format on October 20, 2019, from UniprotKB; http://www.uniprot.org/; 17,546, 3,867, 12,437, and 208 entries, respectively). The sequence obtained for the selected protein was subjected to similarity searches through the public NCBI (National Center for Biotechnology) database using the Basic Local Alignment Search Tool (Blast) to confirm the protein identity (Blast, 2020). To determine amino acid sequences, the LC-MS/MS files were loaded into PEAKS Studio software (Bioinformatics Solutions Inc.) and again subjected to analyses in the Uniprot databases.

### Microorganisms and Antimicrobial Activity

Gram-positive and Gram-negative bacteria, fungi, and yeasts were tested for their sensitivity to the chromatographic fractions obtained from the *P*. *patagoniensis* secretion. The bacterial strains used for the tests were ATCC27853 *Pseudomonas aeruginosa* (Gram-negative) and ATCC6633 *Bacillus subitilis* (Gram-positive) from the American Type Culture Collection (ATCC), yeast MDM8 *Candida albicans* from the Department of Microbiology, University of São Paulo (USP), and the fungus *Penicillium expansum* (bread isolated). Microbial inoculum was prepared according to the method recommended by the standard M07-A9 from the Clinical and Laboratory Standards Institute (CLSI, 2012) in bacterial culture and described by NCCLS M38-P (NCCLS, 2002) for fungal inoculums.

The antimicrobial activity was monitored with a liquid growth inhibition assay (de Jesus Oliveira et al., 2019), performed on 96-well sterile microplates with a final volume of 100 µL. Aliquots of 20 µL fractions collected by HPLC were applied to each well, and 80 µL culture medium containing the suspension of a microorganism culture in log phase was added at a concentration of 1 × 10^5^ CFU/ml. Ultrapure water was used as negative control, and the antibiotics Tetracycline and amphotericin B (10 mg/ml) were used as positive controls. Poor broth medium (1.0 g Peptone in 100 ml H_2_O containing 86 mM NaCl, pH 7.2, 217mOsM) was used for bacterial strains, and poor dextrose potato broth (1.2 g potato dextrose in 100 ml H_2_O, pH 5.0,79 mOsM) was used for fungus. Quality control was conducted for the water and the media with aliquots of 100 µL. The microplates were incubated at 30°C, and the bacteria were kept under stirring for 18 h, and the fungi were kept for 36 h without stirring. Microbial growth was evaluated according to the turbidity of the medium by measuring the optical density of the culture in a Victor^3^ (1,420 Multilabel Counter/Victor^3^, PerkinElmer) microplate reader at 595 nm.

The eluted fractions were placed in a liquid growth inhibition assay that inhibited the growth of the tested microorganisms and were screened based on the apparent expression of inhibition (turbidity reduction) and submitted to SDS-PAGE and subsequent mass spectrometry to verify homogeneity and purity.

### MIC and MBC

MIC: After antimicrobial activity was confirmed for some fractions through the initial screening, they were submitted to a test of inhibition of microbial growth in liquid medium to determine the minimum inhibitory concentration (Antimicrobial-peptide-database, 2019). The MIC was performed using a sterile 96-well plate with a final volume of 100 μL. The purified fractions were serially diluted in ultrapure water, beginning with the same volume as the one where the sample showed activity (20 μL), and 80 µL inoculum with the medium solution was added. After 24 or 36 h incubation, the microplate was read at 595 nm absorbance, using a Victor^3^microplate reader (1,420 Multilabel Counter/Victor3, PerkinElmer). MIC was defined as the minimum concentration of the peptide that causes 100% inhibition of the microorganism (Wiegand et al., 2008; Samy et al., 2014). The minimum inhibitory concentration (antimicrobial-peptide-database) is the concentration range between the maximum concentration of the tested peptide in which the microorganism is still growing and is the lowest concentration of the peptide that causes 100% microorganism death (Hetru and Bulet, 1997; Potron et al., 2015).

MBC: To determine the minimal bactericidal concentration (MBC), bacterial growth rates were measured after 96 h at 595 nm (Hancock, 1999; Yamamoto, 2003). The lowest concentration of the fractions that inhibited microbial growth was determined as MBC, indicating 99.5% of the initial inoculum. Microbial growth was monitored by increasing the optical density at 595 nm in a Victor^3^ microplate reader (1,420 Multilabel Counter/Victor^3^, PerkinElmer) modified from O'Driscoll et al. (2013). The assays were performed in triplicates.

### MTT Assay in VERO Cells

The toxicity of patagonin-CRISP was evaluated in mammal cells, i.e., VERO cells (African green monkey kidney cells, *Cercopithecus aethiops*), using MTT assay (Molchanova et al., 2017). Cells were cultured at 37°C in T25 flasks containing 5 ml Leibovitz medium (L-15), supplemented with 10% fetal bovine serum, and kept in a CO_2_ oven until reaching confluence. In the exponential phase, they were suspended in culture medium and counted in a hematocytometer to determine the cell concentration for plating. The cells were seeded in 96-well plates (2 × 10^5^ cells/well) and cultured for 24 h. The molecule was tested with eight two-fold serial dilutions, starting from a concentration of 120 μg/ml, and cells were exposed to the varying concentrations of the molecule for 24 h. After this, 20 µL MTT (5 mg/ml in PBS) was added for another 4 h at 37°C. The formazan crystals were dissolved by adding 150 µL isopropanol, and the plate was incubated at room temperature until all crystals were dissolved. The cytotoxicity results were transformed into percentages in relation to the control (100% viability). For comparison between the treated and control groups, we used the ANOVA parametric.

### Hemolytic Assay

Human red blood cells (hRBCs) were collected from a healthy donor in 0.15 M sodium citrate buffer pH 7.4, washed three times, and centrifuged (700× *g*, 15 min) in 0.15 M PBS (137 mM NaCl, 2.7 mMKCl, Na 2 HPO410 mM, 1.76 mM KH 2 PO 4, and pH 7.4). After the last centrifugation, the erythrocytes were suspended in PBS, pH 7.4.50 µL (120 μg/ml), and protein aliquots were tested in duplicate by adding 50 µL3% (v/v) solution of human erythrocytes in PBS on a U-bottom plate and incubated for 3 h at 37°C. The supernatant was transferred to a new flat-bottom microplate and used to determine the hemolytic activity of the protein with absorbance measurement at 405 nm in a Victor3 (1,420 Multilabel Counter/Victor3, PerkinElmer) plate reader. The percentage of hemolysis was expressed in relation to 100% of the lysis control (erythrocytes incubated with 0.1% triton X-100); PBS was used as a negative control.

## Results

### Fractionation of the Molecules from Durvenoy's Gland Secretion

After being extracted, Duvernoy's secretion was processed and submitted (2 mg/ml) to a fractionation step on a semipreparative C18 Júpiter® HPLC apparatus. In all, 80 fractions were eluted by the linear gradient of ACN (0–80%). All eluted molecules were tested against the microorganisms in a liquid growth inhibitory assay. Three fractions with antimicrobial activity were displayed (2, 31, and 33) (Table 1).

**TABLE 1:** Antimicrobial activity fractions from Duvernoy's gland secretion.

ACN%	Fraction	Microorganism			
		*Pseudomanas aeruginosa*	*Bacillus subitilis*	*Candida albicans*	*Penicillium expansum*
		ATCC27853	ATCC6633	MDM8	(Bread isolated)
5	2	+	−	−	−
52	31	+	−	−	−
55	33	+	+	−	+

**FIGURE 1:**
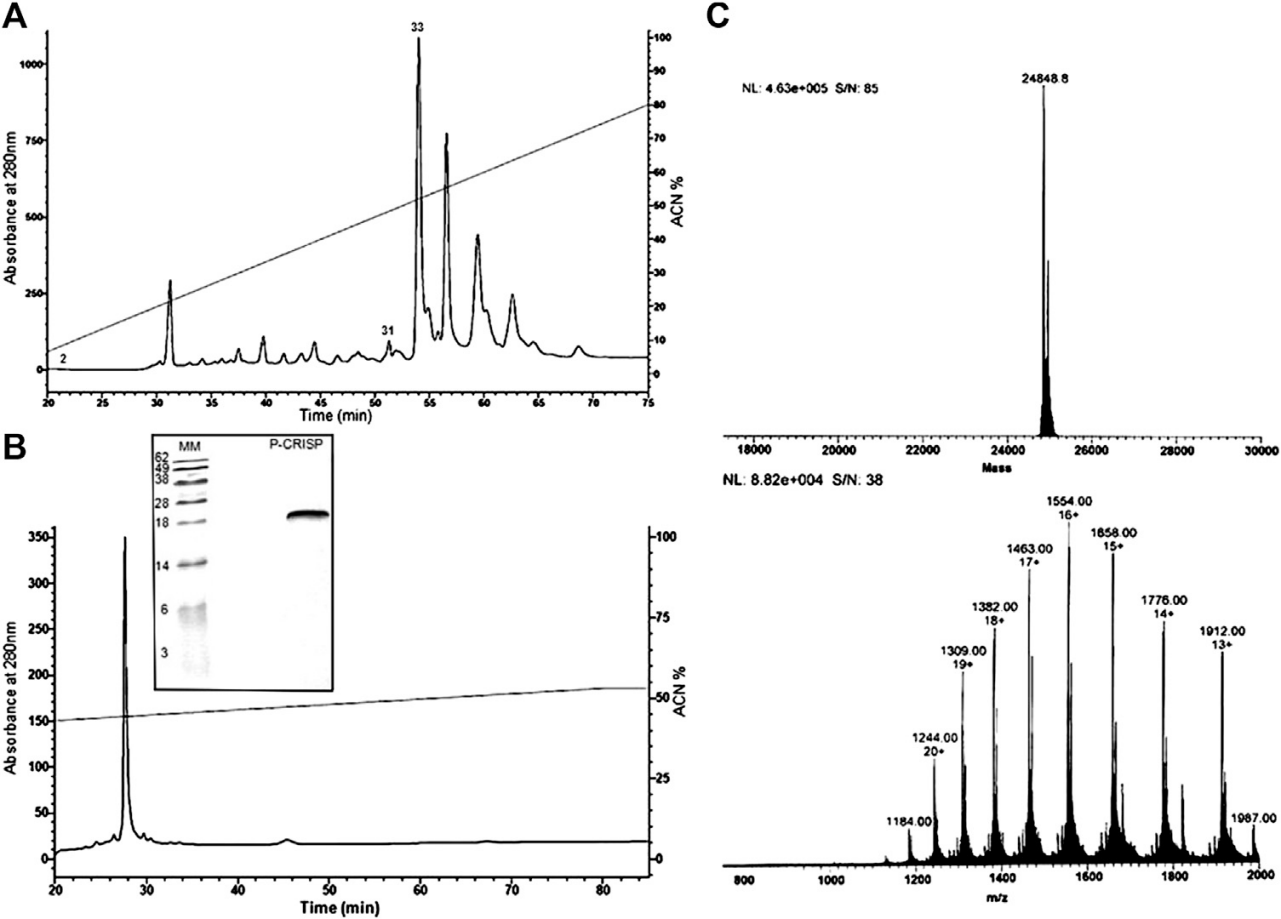
Purification steps and molecular mass characterization. **(A)** Chromatographic profile of Duvernoy's gland secretion. Sample was subjected to a Jupiter^®^ C18 semipreparative column (10 μm, 300 Å, 10 × 250 mm, Phenomenex™), with a 0–80% linear gradient of ACN in acidified water for 80 min at 2 ml/min flow rate. Absorbance was monitored at 280 and 225 nm. The fractions indicated by the numbers (2, 31 and 33) had antimicrobial activity. **(B)** Second step of purification by RP-HPLC. The chosen fraction (33) was subjected to an analytical column Jupiter^®^ C18 (10 μm, 300 Å, 4.60 mm × 250, Phenomenex™) to check the homogeneity. The prominent peak was hand collected and submitted to a test of the inhibition of microbial growth in a liquid medium and mass analyzing. The SDS-PAGE analysis under reducing conditions shows a single band, with a mass of 25 kDa approximately, and confirms the fraction homogeneity. **(C)** Mass spectrometry analyses. The deconvolution of the ions revealed an m/z of 24,848.8 Da. The spectrum was submitted to the MagTran^®^ software.

The fraction eluted with ≅55% CAN (fraction 33) was chosen for the characterization (Figure 1A). This fraction was active against *Pseudomonas aeruginosa* (ATCC27853), *Bacillus subitilis* (ATCC6633), and *Penicillium expansum* (isolated from bread) at a concentration of 240 μg/ml. Fraction homogeneity was assessed using RP-HPLC in an analytic column Júpiter® C18 LC-Column (Figure 1B). The fractions collected were retested, and the antimicrobial activity of the major peak was reconfirmed by the inhibition of microbial growth in a liquid medium.

The molecule was applied (20 µg) to SDS-PAGE 20% polyacrylamide under reducing conditions, where the mass was compared with the molecular weight marker (Life Technologies®). In the gel, the homogeneity of the sample was confirmed (Figure 1B), and the molecular mass obtained was between 28 and 18 kDa.

### Protein Identification

**FIGURE 2:**
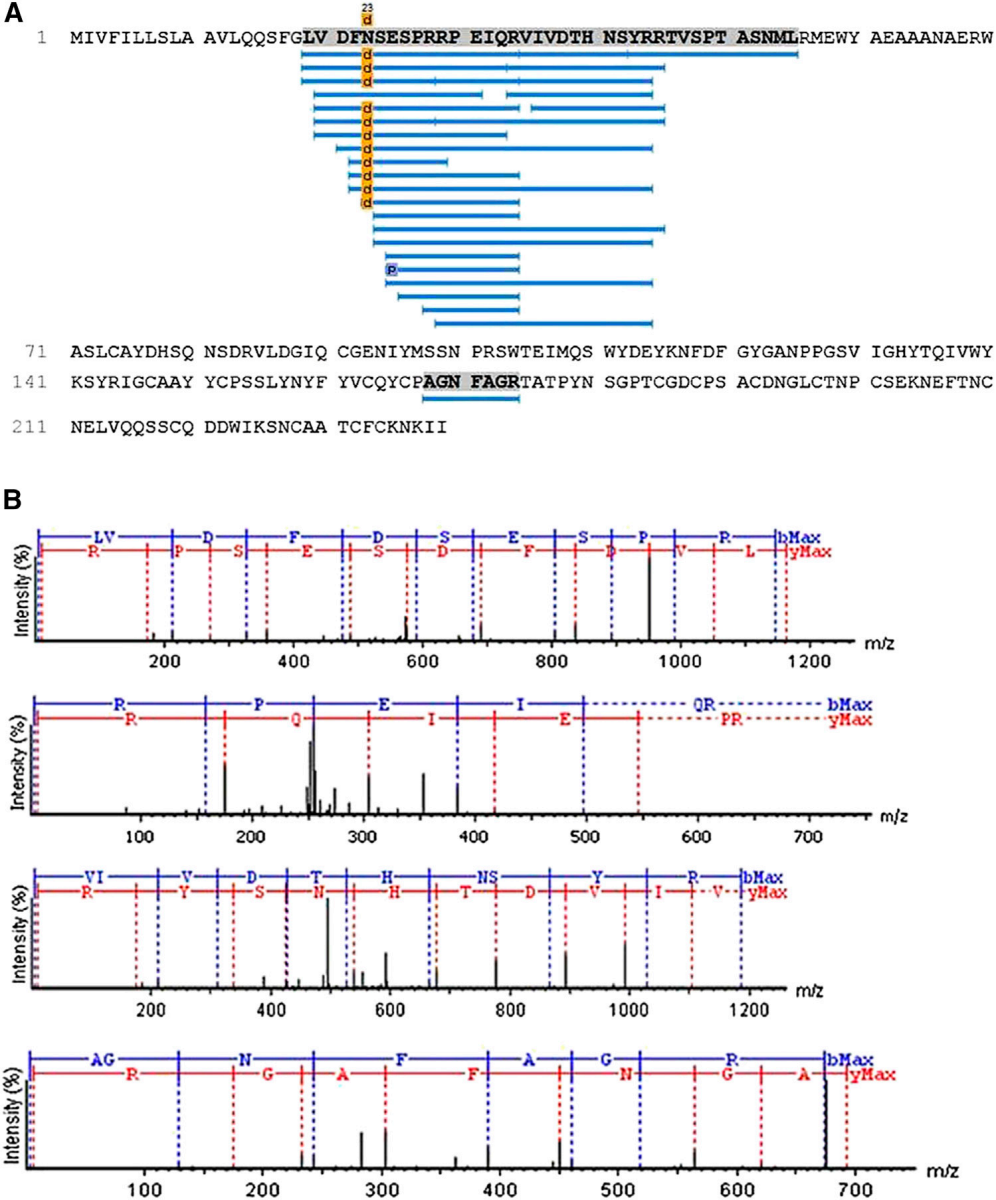
Patagonin-CRISPidentification. **(A)** Through PEAKS Studio analyses with the Uniprot database, a cysteine-rich venom protein from *Philodryas olfersii* (AAZ75603.1) was identified by the presence of some unique fragments in our molecule. **(B)**The unique peptide fragments obtained with 99% ALC. The ions belonging to the y (red) and b (blue) series, indicated in the spectrum, correspond to the amino acid sequences of the peptide LVDFDSESPRRPEIQRVIVDTHNSYRR and AGNFAGR.

Aliquots of molecules digested and not digested by trypsin were analyzed separately by mass spectrometry in an LC-MS/MS, coupled with an LTQ-Orbitrap Velos. The analyses of the undigested molecule by MagTran® revealed a single protein with a mass (m/z) of 24,848.8 Da (Figure 1A), which corroborated the results obtained in SDS-PAGE analyses. Analyses of digested sample spectra with PEAKS Studio were used to compare the spectrum with Uniprot databases; keeping in mind that a unique peptide is defined as a peptide that exists only in one protein of interest, irrespective of length (Zhao and Lin, 2010), we identified a CRVP from *Philodryas olfersii* (AAZ75603.1) through the presence of unique fragments in our molecule (Figure 2A).

The analyses performed using PEAKS Studio allowed us to determine a peptide fragment of a primary structure of patagonin-CRISP, obtained with 99% average local confidence (ALC) from the N-terminal region, composed of 26 amino acids (LVDFDSESPRRPEIQRVIVDTHNSYRR). Another unique short fragment belonging to the C-terminal region has also been identified, and PEAKS Studio revealed a sequence composed of seven amino acids (AGNFAGR) (Figure 2B).

The N-terminal sequence was submitted to BLAST analyses using the BLAST protein function (BLASTp), which found a 100% identity and high reliability index with different fragments of CRVPs (sequence IDs: AJB84505.1, Q09GJ9.1, AAZ75604.1, P86537.1, P0MG5.1, and others). One of these is patagonin (P85099.1), a CRISP from Duvernoy's gland secretion of *P*. *patagoniensis* that was previously identified by Peichoto et al. (2009) and deposited in the database (Peichoto et al., 2009).

### Antimicrobial Assays

**FIGURE 3:**
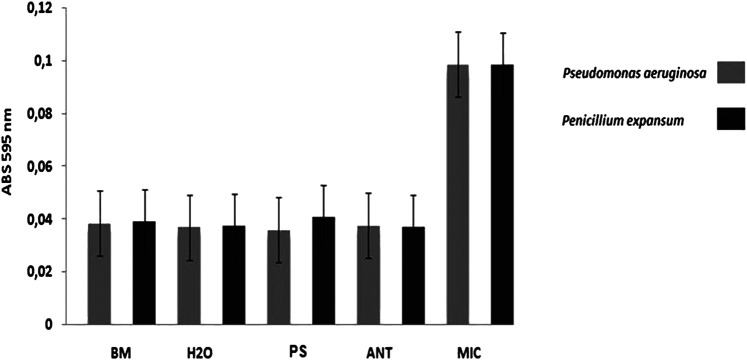
Antimicrobial activity. In the graph it is possible to compare the results of the antimicrobial activity presented by the treatments with the samples in comparison to the controls. Result of antimicrobial assays against *Pseudomonas aeruginosa* ATCC27853 (MIC 1 –**in grey**) and *Penicillium expansum* (MIC2 –**in black**) by reading absorbance at 595 nm. **BM** –Broth medium (PB to bacteria and PDB to fungus): 100 μl; H2O –sterile ultrapure water: 100 μl; **MIC** –microorganism: 20 μl of sterile ultrapure water + culture medium with inoculums; **ANT** –antibiotics (Tetracycline to bacteriaand Anfotericin B to fungus): 20 μl ofantibiotic (10 mg/ml) + culture medium with inoculums; **PS** –protein sample (0.3 μM for **MIC 1** and 0.15 μM for **MIC 2**) in sterile ultrapure water: 20 μl of sample +culture medium with inoculums. **Obs.**: Microorganisms shown in sterile microplates with a lid. The bacteria, being aerobic organisms, were kept under agitation. The incubation period was 18 h for bacteria and 36 h for fungus, at 30°C (Orbital Shaker for Microplates).

The antimicrobial activity of the native protein called patagonin-CRISP was screened against Gram-negative and Gram-positive bacteria, yeast, and fungus strains. The protein was active against *Pseudomonas aeruginosa* (ATCC27853) and *Penicillium expansum* (isolated from bread). The MIC of patagonin-CRISP was 15–7.5 μg/ml (0.6–0.3 µM) and 7.5–3.7 μg/ml (0.3–0.15 µM). The MBC was 30–15 μg/ml (1.2–0.6 µM) against both microorganisms (Table 2). The results of the absorbance readings of the plates are shown in Figure 3. To confirm the antimicrobial activity of patagonin-CRISP again, the eluted protein from the gel was retested at a certain concentration only against *Pseudomonas aeruginosa* (ATCC27853), and it exhibited its effects at 120 μg/ml (4.8 µM).

**TABLE 2:** Minimum inhibitory concentration and minimum bactericidal concentration.

Microorganism	MIC (µg/ml)	µM	MBC (µg/ml)	µM
*Pseudomanas aeruginosa* (ATCC27853)	15–7.5	0.6–0.3	30–15	1.2–0.6
*Penicillium expansum*	7.5–3.7	0.3–0.15	30–15	1.2–0.6

MIC reports the minimum inhibitory concentration that is required to achieve 100% inhibition of microbial growth; MBC refers to the minimum inhibitory concentration that inhibits microbial growth after 96 h.

### Cytotoxicity

**FIGURE 4:**
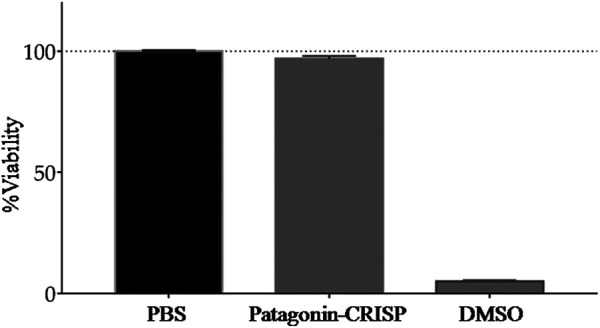
Cytotoxicity analysis. MTT assay analysis of patagonin-CRISP against VERO cell line did not demonstrate significant cytotoxicity at <120 μg/ml concentrations.

Cells from the Vero line were used to test the cytotoxicity of patagonin-CRISP. Protein cytotoxicity was evaluated at the same concentration as the antimicrobial assay, and no sign of cytotoxicity was observed with patagonin-CRISP in the concentration range of 3–120 μg/ml. Viability was approximately 97% after treatment, and no morphological changes were observed. The obtained data were analyzed statistically with analyses of variance (Molchanova et al., 2017), followed by the Bonferroni post-test, executed in the Graph Pad Prism 5.0 software. The compiled results are presented in Figure 4.

Hemolytic activity was evaluated at a concentration of 120 μg/ml, the maximum concentration at which antimicrobial activity was tested, and no hemolytic activity was identified for human red blood cells (results not shown); this suggests that at other concentrations (<120 μg/ml), it is not toxic.

Higher concentrations (>120 μg/ml) have not been tested due to the small amount of native protein available.

## Discussion

CRISPs are widely distributed in reptile venom (Mosmann, 1983; Utkin, 2015) and exhibit a wide range of activities. However, the biological activity of most CRVPs has not been described, and their roles in envenomation remain unknown (Osipov et al., 2005; Levinskaite, 2012; Adade et al., 2014; Samy et al., 2014). In this work, we found a CRISP with antimicrobial activity from Duvernoy's gland secretion of the *Philodryas patagoniensis* snake.

Using RP-HPLC and LC-MS/MS, we found a protein with a molecular mass of 24,848.8 Da or the mass range presented by CRISPs, which usually have weights of about 20–30 kDa (Eberspaecher et al., 1995; Osipov et al., 2005). CRISPs are a group of nonenzymatic, single-chain proteins with homologous amino acid sequences, containing 16 highly conserved cysteine residues in the C-terminal region, forming eight disulfide bridges. These features distinguish this family of proteins, which also has two distinct functional domains: C-terminal, that is, a cysteine-rich region, which includes an ion-channel regulation domain (Gibbs et al., 2008), and N-terminal region, which combines to form the CRISP, antigen 5, and PR-1domain (Gibbs et al., 2008).

The CRISP we identified using PEAKS Studio was found to be from *Philodryas olfersii* (AAZ75603.1) due to the presence of some unique fragments in our molecule. According to Zhao and Lin (2010), a fragment is defined as a single peptide that exists only in one protein of interest and that peptide may appear more than once in the protein's amino acid sequence. The analyses of patagonin-CRISP spectra obtained from PEAKS Studio led us to determine a peptide with 99% ALC. This peptide belongs to the N-terminal region of CRISP and is composed of 26 amino acids in the sequence LVDFDSESPRRPEIQRVIVDTHNSYRR. A unique short peptide belonging to the C-terminal region was also identified, and it is composed of seven amino acids (AGNFAGR). We decided to focus on the N-terminal peptide and submit it to BLAST protein analyses, which found 100% identity with other peptides unique to CRISPs. The application of the concept of unique peptides to the identification of proteins increases confidence in the results.

The amino acid sequence that makes up the N-terminal region characterizes the super-conserved domain of this protein group (Milne et al., 2003; Osipov et al., 2005; Modahl and Mackessy, 2019). One of the CRISPs found by BLASTp was patagonin (P85099.1), a CRVP purified from the venom of *P*. *patagoniensis*. Patagonin has a molecular mass of 24,858.64 Da and a peptide characterized by 14 amino acid residues (VDFDSESPRRPEIQ) (Peichoto et al., 2009). A subtle difference can be observed between the molecular masses of patagonin and patagonin-CRISP, probably due to a variation in the composition of the amino acids.

The composition and the biochemical and pharmacological properties of snake venom vary by species and even between individuals of the same species. Diet, season, age, sex, and habitat are directly related to these possible changes (Koh et al., 2006). Thus, locality differences can lead to species-specific variation in the genetic code and promoting minor changes in the amino acid sequence of proteins but without compromising conserved domains or their functions (Zamudio et al., 2016; Holding et al., 2018).

The domain belonging to the N-terminal is responsible for promoting interaction with the target cell (Milne et al., 2003; Osipov et al., 2005; Gibbs et al., 2008). It is also known that the PR-1 domain is related to antifungal activity and may also have antimicrobial activity, amplifying defense signals via sterols or effectors binding (Niderman et al., 1995; Tadokoro et al., 2020). However, this is the first time that antimicrobial activity has been reported for the CRISP family of proteins.

The toxins in snake venom have molecules of peptide and nonpeptide origin and with wide chemical and functional variability, and they have evident richness in bioactive compounds. Previous findings indicate that proteins isolated from snake venom can lead to the production of clinically and commercially viable drugs. For example, we have the antihypertensive Captopril, using a peptide extracted from *Bothrops jararaca* venom, and the antiplatelet agents Integrilin (eptifibatide) and Aggrastat (tirofiban), taken from the venom of *Sistrurus miliarius barbouri* and *Echis carinatus*, respectively (Cushman and Ondetti, 1991; Koh and Kini, 2012; Kini and Koh, 2020).

Other drugs are already under development, such as VRCTC-310-Onco, composed of two toxins, a crotoxin isolated from the poison of the *Crotalus durissus terrificus* and a cardiotoxin isolated from the poison of the *Naja atra* (Calderon et al., 2014)*.*

Many studies have shown the antimicrobial potential of snake venom and their components (Perumalsamy et al., 2014). The venom of snakes from the families Viperidae and Elapidae has received the most exploration in the search for new antibiotics and exhibit a broad spectrum of activity against different microbial strains. At present, research is focused on combating resistant microorganisms that pose a threat to both public health and the economy (Bocian and Hus, 2020).

Patagonin-CRISP was screened against various strains of microorganisms with liquid growth inhibition assay. The microbial strains chosen are drawn from snakes’ oral cavities or are of medical and economic relevance (Hetru and Bulet, 1997; Vassilara et al., 2017; Yin et al., 2017; Ghosh et al., 2018; Yadav et al., 2018).

The molecule acted against *Pseudomonas aeruginosa*, Gram-negative bacteria, and *Penicillium expansum*, a filamentous fungus. Four microbial strains were chosen to perform this screening to identify antimicrobial activity for the native protein. Because venom yields from rear-fangs snakes can be very low and we were using a native protein (Modahl and Mackessy, 2019), this provides a certain limitation on production until a synthetic protein is available.

The MIC obtained for patagonin-CRISP in this study suggested a potent activity with low concentrations (0.6–0.3 µM against *P. aeruginosa* and 0.3–0.15 µM against *P*. *expansum*). The same was true for MBC, where the protein showed activity at concentrations below 1.2 µM, which means that a low quantity of the protein would be required to inhibit microbial growth; further, it does not show cytotoxicity and hemolytic activity at the concentrations tested. This result highlights the advantages of exploring the protein here identified, in view of the need for new effective substances, that can fight bacterial species that become resistant after a few days or even hours of exposure (Niderman et al., 1995; Overbeck et al., 2017; Gomes et al., 2018).

The identification of new antimicrobial agents originating from venom, which present different targets of action, together with the search for new routes of administration, makes this a very promising line of research (Bocian and Hus, 2020).

The mode of action of patagonin-CRISP remains unclear, but it is known that some CRVPs have already been characterized to inhibit several ion channels, which suggest that patagonin-CRISP might recognize and act on certain membrane targets (Martinac et al., 2008; Modahl and Mackessy, 2019). Our next step will be to express and obtain recombinant protein to allow it to be tested on a larger microbial spectrum so that we can investigate the mechanism of action of patagonin-CRISP.

Natural products are promising candidates against multidrug-resistant pathogens and for the suppression of biofilm formation due to their activities and low toxicity. They also can help the entry of other substances into cells, such as antibiotics, and act as antiresistance compounds to classic antibiotics (Dosler and Karaaslan, 2014).

## Conclusion

These results indicate a new function of a CRVP and indicate a pharmacologically relevant substance for exploration. This article reports the identification of the first CRISP from *Philodryas patagoniensis* Duvernoy's gland secretion with antimicrobial activity against Gram-negative bacteria and filamentous fungus.

## Data Availability Statement

The datasets presented in this study can be found in online repositories. The names of the repository/repositories and accession number(s) can be found in the following: ProteomeXchange PRIDE repository, accession no: PXD022842.

## Ethics Statement

The use of animals in this study was approved by the Butantan Institute Animal Ethics Committee, protocol number CEUA9997010416, Biodiversity Authorization and Information System (SISBIO) no 53845-1 and approved by the São Paulo Zoological Park Foundation through the craft number 181/2016/FPZSP, under the project registration no 385.

## Author Contributions

JB, PS, and RM were responsible for conceptualization; JB was responsible for data curation; JB, AD-R, and PS were involved in formal analysis; PS and RM were responsible for funding acquisition; JB performed investigation; JB, MT, PS, and RM were responsible for methodology; PS was involved in project administration; JB, AR, and PS were responsible for resources; PS and RM were involved in supervision; JB and PS were involved in validation; JB wrote the original draft; JB, AR, MT, PS, and RM wrote, reviewed, and edited the manuscript.

## Funding

This research was funded by the Research Support Foundation of São Paulo (FAPESP), grant no. 2016/24867-1; FAPESP/CeTICS, grant no. 2013/07467-1; Brazilian National Technological and Scientific Development Council CNPq, grant no. 472744/2012-7.

## Conflict of Interest

The authors declare that the research was conducted in the absence of any commercial or financial relationships that could be construed as a potential conflict of interest.
